# A TRANSDISCIPLINARY APPROACH TO THE TREATMENT OF DRY EYE DISEASE IN PATIENTS WITH PSYCHIATRIC DISORDERS

**DOI:** 10.1192/j.eurpsy.2023.1599

**Published:** 2023-07-19

**Authors:** I. Bakija, M. Bogadi, M. Tripković, S. Kaštelan

**Affiliations:** 1 Department for Integrative psychiatry, Psychiatry Clinic Sveti Ivan; 2 Hospital for child and adolescent psychiatry; 3 University Hospital Centar Zagreb; 4 Department of ophthalmology, Clinical Hospital Dubrava, Zagreb, Croatia

## Abstract

**Introduction:**

Dry eye disease (DED) is a multifactorial disease of the tear film and ocular surface representing one of the most common problems in ophthalmological practice and significant public health problem. Characteristic symptoms of DED include gritty, sandy foreign body sensation as well as visual disturbances that have a negative impact on the patient’s daily activities and also affecting patient’s quality of life (QOL).

**Objectives:**

The objective of this research is to point out the importance of transdisciplinary approach to treatment of dry eye disease in patients with psychiatric disorders.

**Methods:**

We reviewed all current available literature in Pubmed dealing with the topic of connection of dry eye disease and psychiatric disorders.

**Results:**

In recent years, the relationship between DED and psychiatric disorders has been gaining attention. A number of epidemiological studies have reported a possible association between dry eye and psychiatric disorders showing that the subjective symptoms of dry eye can be affected not only by changes of the tear film and ocular surface but also psychological factors such as anxiety, depression, schizophrenia, post-traumatic stress disorder (PTSP) and subjective happiness. Apart from psychiatric disorders, psychiatric medications are also considered as risk factors for DED due to their influence on the tear film status. The incidence of ocular side effects increases rapidly with the use of polypharmacy, a very common form of treatment used in psychiatry.

Mental health disorders may be one of considerable contributing factors for dry eye symptoms and undiagnosed mental health conditions can be an influencing element for unexplained levels of DED symptoms. Depression, anxiety, stress, hypochondriasis, neuroticism, sleep and mood disorders may be associated with the exacerbation of symptoms to degrees that are not consistent with the objective signs related to tear dysfunction as well as changes in the anterior surface of the eye.

There is often inconsistency between signs and symptoms of DED, where symptoms often are more related to non-ocular conditions including psychiatric disorders than to tear film parameters. Consequently, in many cases DED may be considered as a psychiatric as well as ophthalmological problem. Psychiatrists and ophthalmologists need to be aware of the potential influence of psychiatric disorders and medications on tear film stability.

**Conclusions:**

A detailed medical history, thorough ophthalmological examination and referral to a psychologist or psychiatrist may be essential in the treatment of those patients. In treatment of psychiatric patients, an integrative and transdisciplinary approach will result in better functioning and higher QOL.

**Disclosure of Interest:**

None Declared

